# Regulation of the Mammalian SWI/SNF Family of Chromatin Remodeling Enzymes by Phosphorylation during Myogenesis

**DOI:** 10.3390/biology9070152

**Published:** 2020-07-03

**Authors:** Teresita Padilla-Benavides, Pablo Reyes-Gutierrez, Anthony N. Imbalzano

**Affiliations:** 1 Department of Biochemistry and Molecular Pharmacology, University of Massachusetts Medical School, Worcester, MA 01605, USA; pablo.reyesgutierrez@umassmed.edu; 2 Department of Molecular Biology and Biochemistry, Wesleyan University, Middletown, CT 06459, USA

**Keywords:** myogenesis, chromatin remodeling enzymes, cell signaling, SWI/SNF

## Abstract

Myogenesis is the biological process by which skeletal muscle tissue forms. Regulation of myogenesis involves a variety of conventional, epigenetic, and epigenomic mechanisms that control chromatin remodeling, DNA methylation, histone modification, and activation of transcription factors. Chromatin remodeling enzymes utilize ATP hydrolysis to alter nucleosome structure and/or positioning. The mammalian SWItch/Sucrose Non-Fermentable (mSWI/SNF) family of chromatin remodeling enzymes is essential for myogenesis. Here we review diverse and novel mechanisms of regulation of mSWI/SNF enzymes by kinases and phosphatases. The integration of classic signaling pathways with chromatin remodeling enzyme function impacts myoblast viability and proliferation as well as differentiation. Regulated processes include the assembly of the mSWI/SNF enzyme complex, choice of subunits to be incorporated into the complex, and sub-nuclear localization of enzyme subunits. Together these processes influence the chromatin remodeling and gene expression events that control myoblast function and the induction of tissue-specific genes during differentiation.

## 1. Introduction

### 1.1. The Family of SWItch/Sucrose Non-Fermentable (SWI/SNF) Chromatin Remodeling Enzymes

Chromatin remodeling enzymes use the energy of ATP hydrolysis to alter histone:DNA contacts in nucleosomal DNA to modify the position or to disrupt the structure of nucleosomes. The consequences of these changes include permitting or restricting access of DNA binding regulatory factors to the chromatin, which can lead to changes in the ability of these factors to regulate DNA-templated processes such as transcription, replication, repair, and recombination. Most chromatin remodeling enzymes are multi-subunit complexes consisting of an ATPase and auxiliary proteins that help specify function.

Several families of chromatin-remodeling complexes have been described to date. These include the SWI/SNF, ISWI, INO80, and CHD complexes [1,2,3,4,5,6,7]. Components of the SWI/SNF (SWItch/Sucrose Non-Fermentable) complex were initially discovered in *Saccharomyces cerevisiae* as essential for transcription of genes involved in the mating-type switching and sucrose fermentation pathways [8,9,10]. Some of these SWI and SNF gene products were shown to form a multi-subunit complex that worked to oppose the repressive effects of chromatin [11,12]. Subsequent in vitro work demonstrated that purified SWI/SNF complexes from yeast and human cells altered nucleosome structure in an ATP-dependent manner and, as a consequence, facilitated nucleosome binding by transcription factors (TFs) [11,12,13,14,15]. Conclusive evidence of catalysis by SWI/SNF complexes followed [16].

In *Drosophila melanogaster*, a complex called BAP, which is homologous to the yeast SWI/SNF complex, is required for expression of homeotic and segmentation genes and regulates the wingless signaling pathway [17,18,19,20,21]. These functions are essential for development since deletion of BAP subunits during embryogenesis is lethal [22,23]. Not surprisingly, depletion of many of the mammalian SWI/SNF (mSWI/SNF) subunits in mice also results in embryonic or peri-natal lethality [23,24].

In mammalian cells, the first evidence for SWI/SNF enzyme function showed a role as a cofactor for transcriptional activation by nuclear hormone receptors [25,26,27]. Subsequent studies demonstrated that its co-activator function was mediated by direct chromatin remodeling activity on integrated viral genes or endogenous cellular genes [28,29]. Additional studies implicated the mSWI/SNF chromatin remodeling enzyme activity in promoting transcriptional repression, as well as other DNA transactions [30,31,32,33,34,35,36].

The mSWI/SNF complex is present in multiple divergent forms [13,15,37,38,39,40]. Two homologous, mutually exclusive ATPases, called BRM (Brahma) and BRG1 (Brahma related gene 1) can act as the catalytic subunit for mSWI/SNF enzyme complexes [25,26,27]. At least 11 additional subunits, or BRG1/BRM-associated factors (BAFs), are structural and functional components of the mSWI/SNF enzyme complexes. Many of these subunits are orthologous to subunits of yeast SWI/SNF and *Drosophila* BAP complexes [25,26,41,42,43]. The existence of splice variants, multiple isoforms of some of the subunits, and tissue-specific gene expression results in the possibility of hundreds of different potential combinations of proteins in any given enzyme complex, and it is widely believed that the combination of subunits that are present determines the functional specificity of the enzyme [44,45]. Recent efforts have significantly advanced understanding of enzyme complex assembly by demonstrating step-wise assembly of three main sub-families of mSWI/SNF complexes [40]. The links between enzyme composition and function, however, remain poorly understood.

Subunit composition is not the only mechanism by which chromatin remodeling enzyme function can be regulated. Post-transcriptional modifications such as acetylation, methylation, sumoylation, phosphorylation, among others, modulate the activity of the mSWI/SNF complex [46,47,48,49,50,51,52,53,54]. Signaling pathways involve a fine-tuned, differential regulation of kinases and phosphatases that are essential for lineage determination and tissue development and maturation [55,56,57]. Protein kinases and phosphatases direct cell fate through the reversible processes of phosphorylation and dephosphorylation, respectively. Hundreds of these enzymes and specific targets are known in higher eukaryotes, and have been reviewed elsewhere [58,59,60,61]. However, our understanding of regulated phosphorylation to control chromatin remodeling processes in the context of lineage determination and differentiation is limited. Emerging evidence has shown that signal transduction pathways are involved in the phosphorylation of subunits of the mSWI/SNF complex, modulating its catalytic activity and its cofactor function during tissue differentiation. Here we summarize work examining modulation of phosphorylation of mSWI/SNF subunits that functionally impacts myoblast proliferation and differentiation.

### 1.2. Myogenesis

The development of skeletal muscle is a complex, multi-step process in which mesoderm-derived structures form somites that then serve as the source for all skeletal muscles in the body. Somites are transient paired structures that align on either side of the neural tube around day 8 of mouse embryogenesis. Somitic cells will commit to specific lineages, including skeletal muscle, due to the influence of signaling molecules produced around the periphery [62,63,64,65,66,67,68]. Initial events give rise to the embryonic or primary muscle fibers of the organism, which lead to the subsequent development of fetal (secondary) fibers that will be the foundation of future adult muscles [65,66,69,70,71,72,73]. Embryonic development of skeletal muscle is established in a multi-step cellular process that involves the activation of gene expression programs to generate the different precursor cell types [62,66,74,75,76]. In mice, skeletal muscle generation begins from embryonic day 8.5 to 9 (E8.5–E9) to E18.5; muscle maturation continues for 2–3 weeks after birth.

Post-natal skeletal muscle stem cells, called satellite cells, exist in a quiescent state under the basal lamina of skeletal muscle fibers and are activated upon muscle injury or hypertrophy signaling. Homeostasis of mature skeletal muscle tissue also requires the activation and mitotic expansion of satellite cells as a mechanism to maintain terminally differentiated myofibers [69,71,77,78,79,80,81,82,83,84]. Maintenance of a functional satellite cell pool relies on a specific transcriptional program. Pax7 is the TF required for maintenance and proliferation of satellite cells [80,82,85,86]. Deletion of *Pax7* in murine models led to death within 2–3 weeks after birth [87]. Mice depleted of *Pax7* have a reduced satellite cell pool, and fail to regenerate muscle [88,89,90].

A wide variety of signaling molecules drive the development of skeletal muscle from embryonic stages throughout post-natal growth [91,92]. Cell signaling events are translated into the activation of myogenic TFs and chromatin remodelers in an organized manner during the maturation of the lineage. The mechanisms by which the cells integrate these signals to define and establish the myogenic lineage are the subject of intense investigation. Here, we will discuss emerging roles for different kinases and phosphatases in the regulation of transcription and chromatin remodeling mechanisms associated with growth and differentiation of the skeletal muscle lineage.

## 2. Cell Signaling Pathways Modulate Chromatin Remodeling Enzyme Function during Skeletal Muscle Myogenesis

Multiple groups determined many years ago that most, if not all, of the mSWI/SNF subunits were phosphoproteins [93,94,95,96]. Despite this knowledge, there has been little progress in determining the functional role of mSWI/SNF subunit phosphorylation, nor has there been significant attention given to the identification of the kinases and phosphatases that generate and regulate phosphorylation. Indeed, the vast majority of our current understanding of mSWI/SNF phosphorylation is limited to modification of the two ATPase subunits, BRG1 and BRM, and of BAF60c, one of three isoforms of the BAF60 protein.

The BRG1 ATPase is essential for viability, proliferation, and differentiation of myoblasts [29,97]. Functional distinctions between the BRG1 and BRM ATPases in the regulation of myogenesis have been identified [98], suggesting non-overlapping roles, but a full understanding of the divergence in the roles of these ATPases is lacking. The BAF60c protein is the BAF60 isoform most highly expressed in skeletal muscle [41], and it is required for normal skeletal muscle development [99]. BAF60 proteins have long been implicated as core mSWI/SNF subunits [40,100], though their molecular role as part of the complex is poorly defined.

### 2.1. The Stress Responsive P38 Kinase Is a Regulator of Chromatin Remodeling Enzymes during Myogenesis

Mitogen-activated protein kinases (MAPKs) phosphorylate the OH- groups of serine/threonine residues to regulate cellular processes such as proliferation, differentiation, survival, and apoptosis. Some members of this kinase family influence the development of skeletal muscle. For instance, extracellular signal-regulated kinase ERK1/2 is critical for myoblast proliferation, and ERK2 is necessary for myocyte fusion [101,102]. ERK activity also promotes the transcriptional activity of MyoD by an undefined mechanism [103]. Maintenance of quiescent satellite cells also relies on the ERK pathway [104].

P38 is the only MAPK that has been demonstrated to directly affect the activity of the mSWI/SNF complex. P38 responds to mitogens as well as a wide range of extracellular stress stimuli [105]. There are four widely expressed, highly homologous p38 isoforms (α, β, γ, and δ). P38α is ubiquitously expressed, whereas the other isoforms show differential expression across tissue types (reviewed in [105,106,107,108]). Over 100 direct targets for p38-mediated phosphorylation have been identified [105,109], demonstrating the broad effects of p38 function. P38 kinases are well-known for regulating pro-inflammatory signaling networks and the biosynthesis of cytokines, such as the tumor necrosis factor-α (TNF-α) and interleukin-1β (IL-1β) in immune cells [110,111,112].

**Figure 1:**
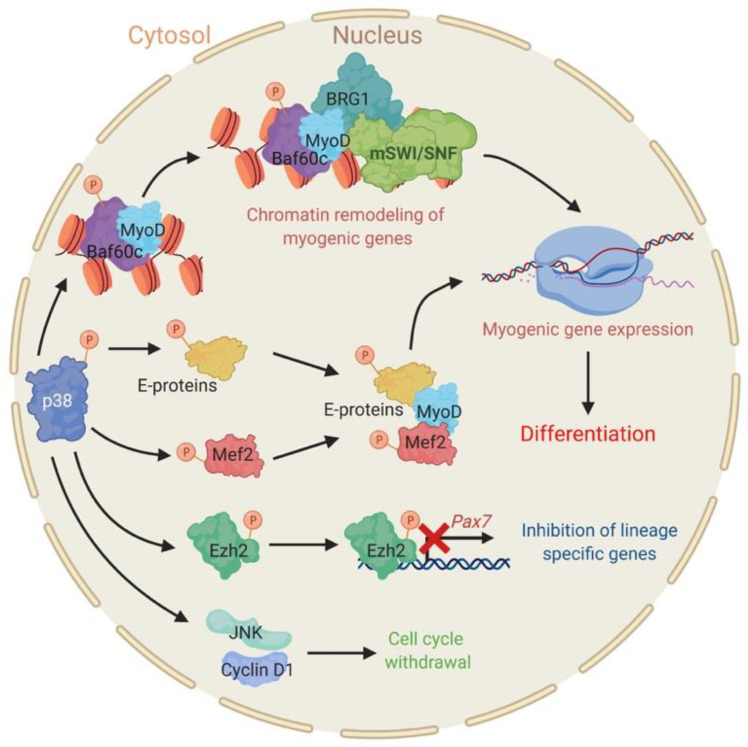
P38 has pleiotropic effects during skeletal muscle differentiation. Phosphorylation of transcription factors (TFs) (E-proteins, Mef2) and the mSWI/SNF subunit BAF60c by p38 enables expression of myogenic genes and differentiation. P38-dependent phosphorylation of Ezh2 downregulates the expression of *Pax7*. P38 also induces cell cycle withdrawal by activating c-Jun N-terminal kinase (JNK) and Cyclin D1.

P38 is a key regulator of skeletal muscle differentiation, with extensive investigation identifying multiple mechanisms of action in satellite cells, in immortalized myoblasts, and in cells of other origins that have been reprogrammed to recapitulate skeletal muscle differentiation [113,114,115,116,117,118,119,120,121,122,123,124]. During myogenesis, there is a sustained increase in the amount of activated p38α and β [125,126,127]. Activation of p38α induces cell cycle withdrawal by upregulating the activity of a different class of MAPK, the c-Jun N-terminal kinase (JNK) and cyclin D1 expression [119]. p38 phosphorylation of the Polycomb group repressor Ezh2 facilitates its interaction with the *Pax7* locus, thereby promoting silencing of this essential satellite cell marker gene [128]. In contrast to functions that inhibit precursor cell properties, p38 also directly promotes myogenic differentiation by phosphorylating E proteins to increasing their dimerization with lineage-determining MyoD and MyoD-related TFs [115], a step necessary for the activation of skeletal muscle-specific gene expression. In addition, p38 phosphorylates MEF2 TFs that cooperate with MyoD family members to activate myogenic gene expression (Figure 1) [122,126,127,129,130,131].

The link between p38 and chromatin remodeling enzyme function derives from work showing that p38 kinase is recruited to myogenic promoters at the onset of differentiation and that its activity is necessary for the recruitment of the mSWI/SNF ATPases BRG1 and BRM to myogenic promoters [114]. These steps are essential for the activation of transcription at muscle-specific genes [29,114,132]. Interestingly, the mSWI/SNF subunit initially identified as the target of p38-mediated phosphorylation was not one of the ATPases but instead the BAF60c subunit [114]. Subsequent efforts showed that the BAF60c subunit, but not the ATPase subunits, formed a pre-assembled complex with MyoD that marks the promoters of myogenic target genes prior to the onset of differentiation signaling. Importantly, knockdown of BAF60c reduced MyoD binding in undifferentiated cells. During myogenesis, p38 phosphorylates the BAF60c subunit at threonine 229, permitting the recruitment of the mSWI/SNF complex ATPases to myogenic promoters and allowing the formation of a functional mSWI/SNF enzyme [114,133]. Chromatin remodeling then permits stable access of MyoD to its cognate binding sites [132] and further recruitment of additional MyoD-associated co-activators (Figure 1) [133]. This work identified function for an mSWI/SNF subunit independent of the ATPase subunits and promoted the idea of physical separation of different mSWI/SNF subunits in myoblasts when biochemical purification of mSWI/SNF enzymes generally have not revealed the existence of sub-complexes [13,14,15,94]. Recent characterization of mSWI/SNF assembly showed that the ATPase subunits are among the last components to be added to the complex [40], which provides further support for this concept. Intriguingly, BAF60c facilitates the recruitment of BRG1 and TFs such as GATA4 to target genes in cardiac progenitors [99,134], but whether phosphorylation by p38 is involved in this process remains to be determined.

The work establishing p38 as a regulator of the mSWI/SNF chromatin remodeling enzymes marked the first evidence that phosphorylation of subunits could contribute to the assembly of the enzyme complex. The work also provided further evidence and support for the idea that myogenic genes are marked for expression in precursor cells, even before the onset of differentiation. Thus, p38 signaling contributes to the activation of gene expression by modulating the activity of chromatin remodelers in addition to regulating TFs.

### 2.2. The Pleiotropic Network of AKT Converges on BAF60c to Promote Myogenesis

AKT, also known as protein kinase B, is a serine/threonine kinase broadly required for cell proliferation and migration, gene expression, and apoptosis [135]. Three isoforms of AKT (1, 2, and 3) have been reported. AKT1 is required for myoblast proliferation but not for differentiation. AKT2 expression and activity increase during differentiation, which promotes myogenesis. AKT2 is not required for myoblast proliferation and is unable to rescue *Akt1* deficiency in proliferating cells [136,137,138,139,140,141]. A recent study has connected the cell adhesion molecule-related downregulated by oncogene (Cdo) and Protein kinase C-related kinase 2 (PKN2) with activation of the AKT signaling pathway [142]. Depletion of Cdo or PKN2 decreased AKT activation and inhibited myogenesis while overexpression of these proteins increased AKT signaling and enhanced differentiation. Studies revealed that regulation of differentiation correlated with the recruitment of BAF60c and MyoD to myogenic promoters [142], though the specific target molecule was not determined. Thus, it remains to be determined whether AKT signaling directly or indirectly affects mSWI/SNF assembly and function and which AKT isoform is responsible. Nevertheless, these data suggest that AKT signaling is influencing the ability of BAF60c to bind to promoters of myogenic genes, whereas p38 signaling activates chromatin-bound BAF60c via phosphorylation. AKT signaling may therefore precede p38 signaling in regulation of mSWI/SNF activity. However, other work using a pharmacological inhibitor showed that BRG1 binding to myogenic promoters was unaffected [143]. The reason for this apparent discrepancy is unclear. It may result from different modes of AKT activation, or the consequences of PKN2 manipulation on BAF60c binding may not be significant enough to preclude BRG1 binding.

### 2.3. Myoblast Cell Cycle Progression and Viability Are Regulated by Casein Kinase 2 (CK2)-Mediated Phosphorylation of BRG1

Recent studies have demonstrated that casein kinase 2 (CK2) phosphorylates BRG1 in proliferating primary myoblasts derived from mouse satellite cells [50,51]. CK2 is a serine/threonine kinase that exists as a tetramer of two catalytic subunits, CK2α or CK2α’, and two regulatory subunits (CK2β). This kinase is ubiquitously expressed and has more than 300 known substrates [144,145,146]. Experiments performed in diverse cultured cell types showed that CK2 inhibition leads to cell cycle inhibition and death [147,148,149,150]. Thus, CK2 has been associated with proliferation, survival, and apoptosis as well as transcriptional regulation of these processes. Additional work links CK2 to lineage determination and differentiation of many tissues [151]. In skeletal muscle, CK2 regulates the activity of Pax3, Pax7, MyoD, and MyoD-related lineage determining TFs [50,152,153,154,155,156,157,158,159]. Studies using immortalized myoblasts showed that each of the CK2 subunits have differential roles in the determination of the skeletal muscle lineage. For instance, CK2α contributes to the activation of the muscle-specific gene program [160], while CK2α’ contributes to the membrane translocation of fusogenic proteins, thus regulating myoblast fusion [160]. CK2β was shown to be essential for MyoD expression in proliferating myoblasts [160].

**Figure 2:**
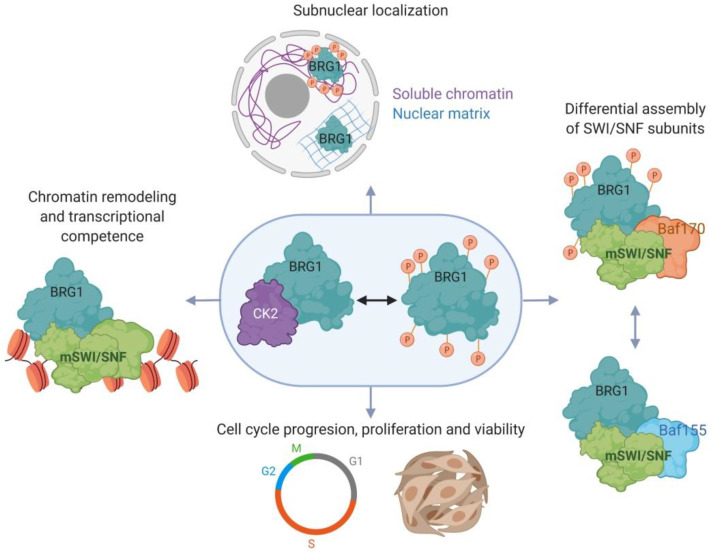
Casein kinase 2 (CK2) modulates mSWI/SNF activity in proliferating myoblasts. CK2-dependent phosphorylation of Brahma related gene 1 (BRG1) regulates the sub-nuclear localization and the subunit composition of the mSWI/SNF complex, viability and cell cycle progression, and the ability to remodel promoter chromatin and promote gene expression that allows myoblast proliferation. The diagram shows the presence or absence of phosphorylation of BRG1 for illustrative purposes. The exact number of phosphorylation sites in the presence of the different mSWI/SNF subunits has not been determined.

In proliferating primary myoblasts, BRG1 is phosphorylated by CK2, and CK2 inhibition or mutation of CK2 target sites in BRG1 resulted in altered myoblast proliferation. Myoblasts expressing a phosphomimetic mutant BRG1 did not proliferate and died. Thus, appropriate regulation of BRG1 phosphorylation by CK2 is required for myoblast survival and normal proliferation. The mechanistic explanation for these results is tied to compromised chromatin remodeling and transcriptional activity of BRG1 at the *Pax7* locus in cells with dysregulated CK2 phosphorylation of BRG1 [50] (Figure 2). Pax7 is an essential transcriptional regulator that maintains proliferation of muscle satellite cells [80,81,85,86]. Deletion of *Pax7* in mice resulted in a decreased number of satellite cells that were progressively lost with aging, and the animals had an impaired ability to regenerate muscle tissue in response to injury [88,89,90,161]. Recent studies demonstrated that the physical interaction between CK2 and BRG1 occurs specifically during mitosis [51]. Confocal microscopy and co-immunoprecipitation analyses showed that CK2 and BRG1 co-localize in cells undergoing mitosis in developing somites of mouse embryos and in primary myoblasts isolated from satellite cells [51]. Importantly, the mitotic CK2-dependent phosphorylation of BRG1 was conserved across different cell lineages.

Phosphorylation of BRG1 by CK2 also contributes to the localization of BRG1 in the nucleus [50]. Sub-nuclear fractionation of proliferating primary myoblasts showed that endogenous BRG1, as well as an ectopically expressed wild type version of BRG1, were associated with both the nuclear matrix and the chromatin [50], consistent with earlier work demonstrating BRG1 can associate with the nuclear matrix [31,162]. Interestingly, a phosphomimetic mutant form of BRG1, was unable to associate with either of these sub-nuclear fractions, and presented higher intra-nuclear mobility, as measured by FRAP, than wild type BRG1 proteins [50]. A mutant version of BRG1 that prevents BRG1 phosphorylation at the CK2 target sites associated only with the nuclear matrix and not with the chromatin fraction. Enzymatic inhibition of CK2 gave the same results. These findings suggest that sub-nuclear BRG1 localization is dynamic and is regulated by CK2 phosphorylation. Whether other mSWI/SNF subunits are similarly regulated in their sub-nuclear distribution remains an open question. Given the hierarchical assembly of mSWI/SNF subunits into functional enzyme complexes [40], sequestration of different subunits in distinct sub-nuclear compartments provides a potential mechanism to achieve regulated assembly (Figure 2).

Another consequence of BRG1 phosphorylation by CK2 is the different subunit composition of the mSWI/SNF complex [50]. BAF155 and BAF170 are structurally related subunits of mSWI/SNF enzymes [29]. Analysis of mSWI/SNF complex assembly and genetic knockout studies indicate BAFs 155 and 170 are essential for the formation of all mSWI/SNF complexes and for the stability of all of the other mSWI/SNF subunits [40,163]. Endogenous BRG1 or an ectopically expressed wild type version of BRG1 preferentially associates with BAF170, while a non-phosphorylatable BRG1 mutant as well as the endogenous BRG1 from myoblasts treated with CK2 inhibitor preferentially interacts with BAF155. A phosphomimetic BRG1 mutant interacted poorly with both BAF170 and BAF155. Thus, CK2 phosphorylation of BRG1 contributes to the regulation of the subunit composition of the mSWI/SNF complex (Figure 2, [50]). Variation in subunit composition has been associated with specialization in the function of the complex [44,45]. For instance, studies using mouse ESCs showed that the expression of the BAF155 subunit is favored over the expression of BAF170, and BAF155 is preferentially incorporated into BRG1-based SWI/SNF complexes. Upon ESC differentiation into the neuronal lineage, expression of BAF155 is downregulated, while BAF170 is induced and incorporated preferentially into the complex [164]. The consequences of incorporating one or the other subunit are not well understood, but presumably relate to specific chromatin remodeling functions at specific loci during developmental or other signaling.

Although there is no direct link to myogenesis, studies of BRG1 and BRM during the cell cycle showed that both ATPases are hyperphosphorylated upon initiation of mitosis and are excluded from the condensed mitotic chromosomes. The levels of BRG1 remained unaffected, whereas the BRM protein may be degraded [93,94]. ERK1, another member of the MAPK family, was implicated as the kinase that phosphorylates BRG1 and BRM and, consequently, inactivates both the ATPase and the chromatin remodeling activities of the mSWI/SNF complex during mitosis [94]. Mitotic condensation of the chromosomes leads to changes in the activities of TFs, cofactors, and RNA polymerase, causing a general repression of transcription and the removal of many of these factors from the chromatin [165,166,167,168,169]. Whether or not phosphorylation of mSWI/SNF ATPases is causal or is a consequence of mitotic chromosome condensation remains to be determined. It also remains to be seen whether evidence supporting or refuting the idea that CK2 and ERK1, the two kinases implicated in modifying the phosphorylation state of the mSWI/SNF complex during cell cycle progression, function independently or in a dependent manner. In either case, it appears that dynamic regulation of BRG1 and mSWI/SNF enzyme phosphorylation and function occurs during mitosis.

### 2.4. The PKCβ_1_ Kinase and the Calcineurin (Cn) Phosphatase Act in Opposition to Regulate BRG1 Phosphorylation and Myogenic Differentiation

**Figure 3:**
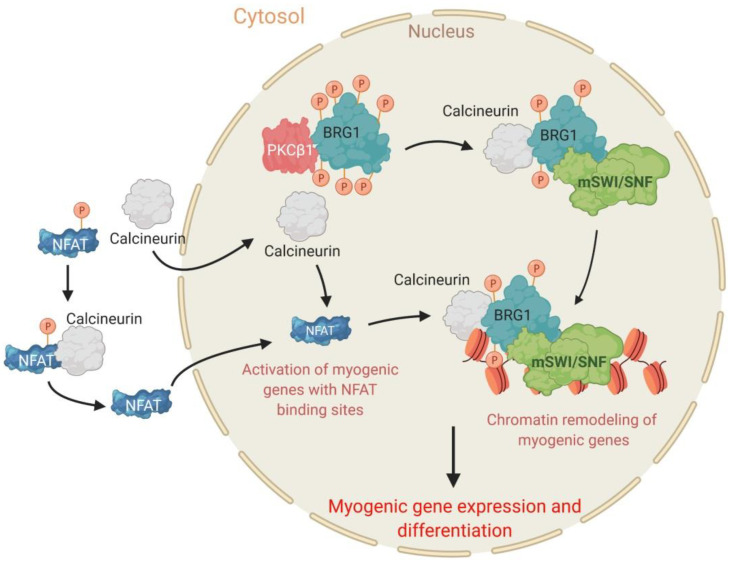
Antagonistic roles of PKCβ_1_ and calcineurin in myogenesis. PKCβ_1_ interacts with and phosphorylates BRG1 to block differentiation. Upon myogenic stimulus, calcineurin dephosphorylates BRG1 and the TF nuclear factor of activated T-cell (NFAT), enabling chromatin remodeling at myogenic loci, myogenic gene expression, and skeletal muscle differentiation.

In the context of skeletal muscle differentiation, a fine-tuned balance of phosphorylation and dephosphorylation of BRG1 driven by PKCβ_1_ and calcineurin controls myoblast fate [49]. It is well known that the calcium-sensitive serine/threonine phosphatase calcineurin [170] contributes to the differentiation of immortalized and primary myoblasts, and for muscle regeneration upon damage in vivo [171,172]. Calcineurin is a heterodimer formed by a catalytic and a regulatory subunit [173,174]. The mechanism of action of calcineurin was characterized in lymphocytes. Active calcineurin dephosphorylates the TF nuclear factor of activated T-cell (NFAT), leading to its nuclear translocation, where it regulates gene expression by binding to promoter regions of target genes [175,176,177,178]. During myogenesis, NFAT binds to target promoters in a calcineurin-dependent manner and contributes to differentiation, the determination of fiber type, the number of primary fibers, and the growth of multinucleated muscle cells (Figure 3) [162,179,180,181,182,183,184]. Calcineurin also triggers skeletal muscle differentiation by mechanisms that are independent of NFAT [185,186]. Other work showed that calmodulin, a calcium-binding second messenger protein, also interacts with the mSWI/SNF complex in other cell types and promotes its chromatin remodeling activities in a calcium-dependent manner [187]. Together, the data supported the hypothesis that the mSWI/SNF complex might be a target of calcineurin.

Studies in primary and immortalized myoblasts demonstrated that calcineurin inhibition blocked chromatin remodeling and expression of myogenic genes without affecting the expression of the lineage determining master regulators for myogenesis, MyoD and Myf5. As a result, differentiation was inhibited [49]. Importantly, calcineurin inhibition also led to hyperphosphorylation of the BRG1 ATPase of mSWI/SNF enzymes (Figure 3). Efforts to identify the kinase that opposes calcineurin function relied on software predictors of kinase activity and screening of inhibitors against those kinases. In a key experiment, inhibition of PKCβ kinases rescued the effect of calcineurin inhibition on myogenic differentiation. The simplest interpretation of such results is that in the absence of PKCβ_1_ activity, there was no consequence of calcineurin inhibition because there were no phosphate groups to remove from the substrate. Knockdown studies identified PKCβ_1_ as the specific isoform responsible. *In vitro* work identified serine residues N- and C-terminal to the bromodomain of BRG1 as targets of PKCβ_1_ and calcineurin [49]. Site directed mutagenesis studies demonstrated that mutation of these sites to phosphomimetic residues prevented myogenesis because the phosphomimetic BRG1 mutant was unable to bind to myogenic promoters while mutation of PKCβ_1_ target sites to non-phosphorylatable (alanine) residues had no effect on chromatin binding or differentiation [49,188]. Calcineurin inhibition also blocked the interaction of other subunits of the mSWI/SNF complex with myogenic promoters [188]. This raises the possibility of regulated dephosphorylation of other mSWI/SNF subunits or an indirect effect due to the failure to dephosphorylate BRG1. The work identifies a kinase and phosphatase working in opposition to each other to dynamically regulate the activity of a chromatin remodeling enzyme as part of the overall regulatory mechanism governing the initiation of myogenic differentiation (Figure 3).

## 3. Conclusions

**Figure 4:**
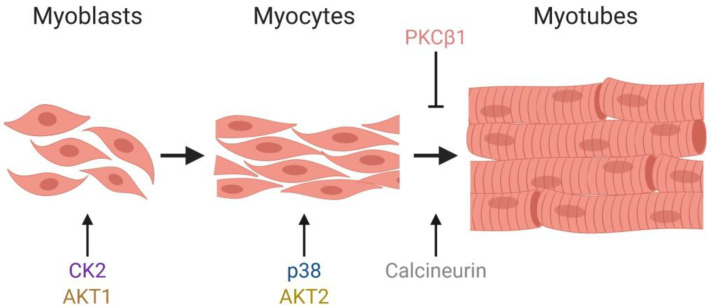
Kinases and phosphatases that modulate the chromatin remodeling activity of the mSWI/SNF complex during myogenesis. Schematic representation of post-natal myogenesis and indications of when specific kinases and phosphatases that control the activity of the mSWI/SNF complex act.

The effect of kinases and phosphatases on mSWI/SNF chromatin remodeling enzymes during myoblast proliferation and differentiation are intriguing and represent another mechanism for cells to regulate differentiation. To date, each characterized pathway has been studied separately, and the crosstalk between these different regulatory signaling remains an important aspect to address. Similarly, evidence for the regulation of mSWI/SNF enzyme function by phosphorylation due to specific kinases exists in contexts other than myogenesis. For example, BAF60c is phosphorylated by PKCζ/λ in response to insulin, resulting in lipogenic gene transcription in liver [52]. BRG1 phosphorylation dependent on the kinase ataxia telangiectasia mutated (ATM) is a mechanism by which the ATPase contributes to double-strand break repair, as the phosphorylation enhances BRG1 binding to γ-H2AX-containing nucleosomes [189]. However, there are a limited number of cell types in which multiple signaling pathways have been implicated in the regulation of mSWI/SNF-dependent processes. Consequently, myoblast proliferation and differentiation represent an exceptional opportunity to take studies of regulated phosphorylation to the next level, which would be to understand how different signaling pathways converge on the same set of target proteins. Whether signaling pathways function independently, are temporally regulated, are dependent on other pathways, or work in a cooperative manner is largely unknown. Given that most, if not all, of the mSWI/SNF component proteins are phosphoproteins, we believe it is highly likely that at least some signaling pathways work in a concerted manner (Figure 4), and that additional signaling pathways affecting mSWI/SNF proteins phosphorylation are yet to be discovered.

In summary, mSWI/SNF enzymes are essential chromatin remodelers that modulate the proper growth and differentiation of mammalian cells. Dysregulation of mSWI/SNF complex activity can lead to consequences in transcriptional regulation, development, and metabolic homeostasis that may progress into severe pathologies such as cancer. Therefore, it is necessary to continue to advance our knowledge of the regulatory events that control the physical and functional states of individual subunit proteins and the enzyme complex. Reversible cell signaling events that cause phosphorylation and dephosphorylation play major roles in most aspects of cell and developmental biology. The work summarized in this review demonstrates that regulation of the mSWI/SNF enzyme assembly, chromatin binding, and chromatin remodeling activities are also targets for these kinases and phosphatases (Figure 4). Further dissection of these regulatory mechanisms will reveal insights into the functionality of the mSWI/SNF complex as well as the impact of chromatin remodeling enzyme function on human development and health.
